# Can the AHCL System Be Used in T1D Patients with Borderline TDDI? A Case Report

**DOI:** 10.3390/s21217195

**Published:** 2021-10-29

**Authors:** Anna Tekielak, Sebastian Seget, Ewa Rusak, Przemysława Jarosz-Chobot

**Affiliations:** 1 Students’ Scientific Association at the Department of Children’s Diabetology, Medical University of Silesia, Medyków 16, 40-752 Katowice, Poland; 2 Department of Children’s Diabetology, Medical University of Silesia, Medyków 16, 40-752 Katowice, Poland; sebastian.seget@sum.edu.pl (S.S.); Poland; rusakewa@gmail.com (E.R.); przemka1@tlen.pl (P.J.-C.)

**Keywords:** type 1 diabetes mellitus, borderline total daily dose of insulin, remission, advanced hybrid closed loop systems

## Abstract

(1) Background: Intensive insulin therapy using continuous subcutaneous insulin infusion (CSII) with continuous real-time glucose monitoring (rt CGM) is the best option for patients with T1D. The recent introduction of a technology called Advanced Hybrid Closed Loop (AHCL) represents a new era in the treatment of type 1 diabetes, the next step towards better care, as well as improving the effectiveness and safety of therapy. The aim is to present the case of a T1D patient with a borderline total daily dose of insulin being treated with the Medtronic AHCL system in automatic mode. (2) Materials and Methods: A 9-year-old boy, from October 2020, with type 1 diabetes in remission was connected to the Minimed™ 780G (AHCL) system in accordance with the manufacturer’s recommendations (daily insulin dose > 8 units, age > 7). Records of the patient’s history were collected from visits to The Department of Children’s Diabetology, as well as from the Medtronic CareLink™ software and the DPV SWEET program from October 2020 to April 2021. (3) Results: The patient’s total daily insulin requirement decreased in the first 6 weeks after the AHCL was connected, which may reflect the remission phase (tight glycemic control with a healthy lifestyle). The lowest daily insulin requirement of 5.7 units was also recorded. In a three-month follow-up of the patient treated with AHCL, it was found that for almost 38% of the days the insulin dose was less than 8 IU. (4) Conclusions: The AHCL system allows safe and effective insulin therapy in automatic mode, as well as in patients with a lower daily insulin requirement. The AHCL system should be considered a good therapeutic option for patients from the onset of T1D, as well in the remission phase.

## 1. Introduction

It is a well-known fact of evidence-based medicine that it is extremely important to achieve close to normoglycemic levels to prevent acute and long-term complications of type 1 diabetes (T1D) [1]. Additionally, improving glycemic control without increasing the risk of hypoglycemia presents challenges for both the patient and the clinician. Continuous subcutaneous insulin infusion (CSII) has led to better care for people with diabetes and is currently the best treatment option for patients with T1D [2]. In addition, sensor-augmented pump therapy from the onset of a diagnosis of T1D can lead to better long-term glycemic control and help maintain endogenous pancreatic beta cell function [3].

The recent introduction of a technology called Advanced Hybrid Closed Loop (AHCL) presents a revolution in the care and treatment of T1D. The system shows a reduction in blood glucose fluctuations and the risk of hypoglycemia [4]. AHCL refers to the latest automated hybrid systems. There are three such systems on the world market: Medtronic’s System MiniMed™ 670G [5], and MiniMed™ 780G (SmartGuard™) [6]; Insulet’s Omnipod-Automated mode (HypoProtect™) [7]; and Tandem’s T slim x2 Control IQ [8]. All AHCL systems have restrictions on their total daily dose of insulin (TDDI); they are dedicated to patients with a TDDI of at least 8 [5,9] or 10 [8] units.

In 2020, the MiniMed™ 780G Advanced Hybrid Closed-Loop system was introduced to the world market, which is a development of the SmartGuard™ technology. The system was Conformité Européenne (CE) marked in June 2020 and was introduced to the European market in autumn 2020 [10]. It is now available in at least twelve European countries, and in Poland from the end of December 2020 [11].

AHCL systems are breakthrough devices in the field of diabetes and it is possible that they are a spectacular change for the better—both in maintaining normal blood glucose levels and in improving the quality of life of patients. Another important point is that maintaining normoglycemia allows for better functioning of pancreatic beta cells and, consequently, allows higher concentrations of C-peptide to be maintained for a longer period of time [12].

The authors would like to focus on the above criterion, which is the issue of having a low TDDI. What about patients with so-called “border” values of the TDDI? Will therapy with the MiniMed™ 780G pump in patients with a requirement close to, or less than, 8 units per day affect the parameters related to diabetes control and fulfill its function with equal success?

The aim is to present the case of a T1D patient with a borderline TDDI treated with the Medtronic AHCL system.

## 2. Materials and Methods

### This Work Was Based on the Medtronic Systems

The MiniMed™ 780G system consists of a manual and an automatic mode. The manual system works like the 640G system [13]: it uses SmartGuard™ technology [14] with the introduction of the PLGS (Predictive Low Glucose Suspend) feature. SmartGuard™ is a Medtronic proprietary name that defines automatic insulin dosing based on sensor readings (continuous real-time glucose monitoring (rt CGM)). A new feature is an automatic system with a precise, automatically adjusted dose of basal insulin and automatic correction boluses (every 5 min) as before, based on rtCGM values [9]. SmartGuard™ is designed to keep glucose levels between 70 mg/dL and 180 mg/dL for as long as possible. Therefore, SmartGuard™ has three blood glucose targets: 100 mg/dL, 110 mg/dL, or 120 mg/dL, which should be considered by healthcare professionals in order to determine which SmartGuard™ target value to use to keep glucose levels sustained for as long as possible within an appropriate range [9]. Another progressive step is the possibility of having a therapy partner, who can observe the patient’s blood glucose level at any time via a mobile application [9].

Records of the patient’s history were collected from visits to the Department of Children’s Diabetology, as well as from the Medtronic CGM CareLink™ software and the DPV SWEET program from October 2020 to April 2021.

Glycated hemoglobin HbA1c was routinely performed at the hospital laboratory using HPLC.

## 3. Case Report

A 9-year-old boy with type 1 diabetes was diagnosed at the Department of Children’s Diabetology, Medical University of Silesia in Katowice, Poland, on 14 October 2020. Upon diagnosis, the patient had mild [15] diabetic ketoacidosis (DKA) (venous blood pH: 7.218). He had confirmed positive antibodies, such as anti-GAD: 3405 U/mL (norm < 10 U/mL) and ZnT8: 592.54 U/mL (norm < 15 U/mL). In the first days of treatment, intravenous insulin therapy was started and then subcutaneous treatment was continued using a personal insulin pump—the Medtronic MiniMed™ 640G system (insulin lispro). On 19 February 2021, he was re-admitted to the Department of Children’s Diabetology in order to switch from the MiniMed™ 640G system to the MiniMed™ 780G system. During the transition, the boy learned the principles and the philosophy of the new system and underwent diabetic and dietary re-education by an experienced educator and dietician.

From the beginning of his diabetes, the boy was well controlled and had no episodes of severe hypoglycemia, and DKA was observed. From November, the boy met the criteria for remission. Referring to the remission criteria according to the ISPAD Clinical Practice Consensus Guidelines 2018, partial remission is defined as an insulin requirement of <0.5 units per kg body weight per day and a HbA1c level of <7%. We can conclude that the boy met the remission criteria [16].

In Poland, currently, the 780G system as a new device can be bought privately by the patient, but the entire supply of a personal insulin pump and CGM is partially reimbursed (70%) by the National Health Fund [17] for patients up to 26 years of age.

**Figure 1:**
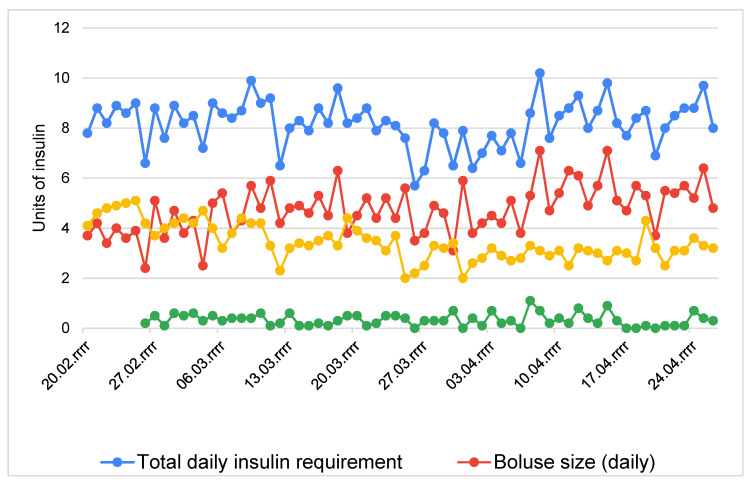
Daily insulin requirement, bolus size, automatic base, and automatic correction per unit of time.

The results of the clinical follow-up of the boy’s AHCL system therapy are presented in Table 1 and Table 2 and in Figure 1.

Table 1 shows the clinical characteristics of the boy during subsequent visits before, during, and after connecting the MiniMed™ 780G system.

The progress of therapy is presented in Table 2, in which we can analyze the rtCGM metrics from the last 15 days of using the Medtronic MiniMed™ 640G system and the first 9 weeks of using the 780G system.

The data in the table show that the patient was in the manual mode for a week, while the automatic mode is shown in the form of biweekly reports. Observation includes 100% use of the sensor.

The patient’s TDDI broken down by bolus size, auto base, and auto correction per unit of time is shown in Figure 1.

The largest corrections were recorded in April and they were 1.1 units, with automatic base at 3.3 units and bolus at 5.3 units and 0.9 units, and with automatic base at 2.7 units and bolus at 7.1 units.

## 4. Discussion

The hybrid pump philosophy has greatly improved glycemic control and quality of life [1]. The introduction of HCL (Hybrid Close Loop) systems has already been a revolution, while AHCL systems are another step in the advancement and improvement of diabetes control. There are not many reports on the AHCL and the 780G system. Additionally, to our knowledge, there have been no reports of patients with a borderline TDDI undergoing such therapy so far.

By analyzing the data in Table 1, it can be seen that the patient was initially overweight [18]. Referring to the ISPAD [19], PTD Diabetes Poland [20], and the WHO’s “recommendations for a healthy lifestyle” [18], a weight loss recommendation was introduced as one important element of the patient’s diabetes therapy. During subsequent visits, we can observe that the patient is within the normal range of BMI values for his age. It should be noted that the patient’s BMI has normalized but he has not reduced his carbohydrate requirement. The boy grew, he lost weight, and thus his insulin requirement changed.

The average daily insulin requirement per kilogram of body weight was initially 0.45 units and was gradually decreasing with subsequent visits, maintaining the target close to normoglycemia. It also meets the first remission criterion. The patient was in remission all the time.

The main focus of our interest is in the TDDI. It can be seen that at the third visit it was 12.5 units, so the patient was eligible to connect to the 780G pump, meeting the criterion of ≥8 units required for AHCL therapy.

Referring to Table 2, the total daily dose of insulin decreased in the first 6 weeks after connecting the MiniMed™ 780G system, which may reflect the remission phase (strict glycemic control). Looking at the fourth pump reading, the lowest TDDI, 5.7 units was recorded. The fifth pump reading shows the lowest average TDDI, 7.5 units, and during this period of 11 days a TDDI of less than 8 units was observed. In a three-month follow-up of the patient being treated with the AHCL system, it was found that for almost 38% of the days the insulin dose was less than 8 IU.

When analyzing the coefficient of variation for glucose (CV), we can observe its reduction, which means lower variability of glycemia during therapy with the MiniMed™ 780G system. Comparing the therapy with 640G and 780G pumps, we can say that the time in range (TIR) slightly improved. Time below range (TBR) fell from 2% to 1%, and was most recently 0%, reflecting a reduction and even elimination of the number of hypoglycemic episodes.

During the therapy in the automatic mode, the bolus size increased. At the same time, the automatic base decreased clearly while adding the automatic correction ranging from 0.3 to 0.4 units.

The authors’ data show that autocorrect appears to improve the TIR [1].

The automatic mode remains at almost 100% and the manual mode only 1% or 0%.

In graph 1, we can analyze data from the period when the patient was using the manual and automatic modes. There are visible drops in the TDDI below 8 units, yet the pump continues to function in the automatic mode.

The data presented in Table 2 and Graph 1 show that with a TDI of <8 units, the MiniMed™ 780G system allows automatic AHCL function.

T1D remission (a low insulin requirement per kg of body weight) reflects partial β-cell regeneration with increased insulin secretion and improved peripheral insulin sensitivity before the insulin production begins to decline [16]. The remission phase begins within days to weeks of starting insulin treatment and may last from weeks to years (rarely). Some studies suggest that for “life with diabetes” patients, early diagnosis of diabetes and treatment targeting normoglycemia may increase the ability to maintain some functions of pancreatic beta cells [21]. Maintaining the function of beta cells reduces the risk of vascular complications and the risk of severe hypoglycemia [16]. The functioning of pancreatic islet cells in patients with T1D is associated with lower HbA1c levels and a reduction in short- and long-term complications [22,23]. It is also known that the use of advanced technology to achieve normoglycemia soon after diagnosis of T1D will be beneficial in maintaining pancreatic beta cell function [24] for a longer period of time.

The main fact is that AHCL systems are becoming an integral part of diabetes care, as they improve glycemic control and reduce fluctuations, while lowering glycosylated hemoglobin (HbA1c) levels and improving quality of life [25]. Moreover, it is also a fact that the implementation of CSII should occur as soon as possible after the diagnosis of the disease to protect the remaining β cells of the pancreas from further destruction in the autoimmune process, thus promoting regeneration and remission [26].

## 5. Conclusions

Taking into account the analyzed results and reports from the literature, it can be concluded that the MiniMed™ 780G system also fulfills its function in patients with a “borderline” TDDI. Therefore, it can be stated that the MiniMed™ 780G system allows safe therapy in an automatic mode, as well as in patients with a low daily insulin requirement, including during the remission phase. We suggest that the MiniMed™ 780G system can also be a good choice and initiated in patients from the clinical start of T1D. The safety of therapy with the AHCL, including a possible manual mode in the system, will be reflected in the patient’s quality of life and better disease control.

The presented case creates a field for reflection on the fact that the AHCL system may be a beneficial therapeutic option for all patients, positively influencing a patient’s quality of life.

## Figures and Tables

**Table 1:** Patient’s clinical data before and after connection to the MiniMed780G pump.

Visit	Date of Visit	Body Weight		Height		Body Mass Index (BMI)		Total Daily Dose of Insulin (TDDI)	Daily Insulin Requirement/kg Body Weight	Glycated Hemoglobin (HbA1c)
		(kg)	(pc)	(cm)	(pc)	(kg/m^2^)	(pc)	(units)	(units/1 kg bw)	(%)
1	02 November 2020	50.90	96	147.5	93	23.60	95	22.70	0.45	12.20
2	16 November 2020	50.90	96	147.5	93	23.60	95	20.02	0.40	5.90
3	17 December 2020	49.00	95	150.00	95	21.80	91	12.50	0.26	5.90
4	19 February 2021	44.70	89	151.50	96	19.50	79	8.50	0.18	5.90
5	18 March 2021	43.40	86	151.50	96	18.90	74	8.20	0.20	6.10

**Table 2:** CGM metrics during therapy with the Medtronic MiniMed780G pump.

Medtronic CGM Metrics							
**Type of System**	MiniMed 640G	Minimed 780G					
	(Last 15 Days of Use)	Manual Mode (1 Week)	Automatic Mode (2 Weeks × 4)				
Period	3 February 2021–17 February 2021	20 February 2021–26 February 2021	27 February 2021–12 March 2021	13 March 2021–26 March 2021	27 March 2021–9 April 2021	10 April 2021–23 April 2021	
Pump read	1	2	3	4	5	6	
Mean glucose concentration from sensor (mg/dL ± SD)	110.00 ± 26	113.00 ± 23	118.00 ± 25	121.00 ± 26	119.00 ± 24	129.00 ± 29	
Glucose management indicator (GMI) (%)	5.94	6.01	6.13	6.06	6.15	6.04	
Coefficient of variation for glucose (CV) (%)	23.84	20.62	20.81	20.64	21.87	21.77	
Time in range (TIR) (%)	96.28	97.67	97.58	98.24	96.39	97.72	
Time above range (TAR) (%)	2	2	2	1	2	3	
Time below range (TBR) (%)	2	1	0	0	1	0	
Mean area under the curve (AUC) > 180 mg/dL (%)	1.78	1.72	1.82	1.63	3.28	1.90	
Mean area under the curve (AUC) < 70 mg/dL (%)	1.94	0.61	0.61	0.13	0.33	0.38	
Mean area under the curve (AUC) < 54 mg/dL (%)	0.00	0.00	0.00	0.00	0.00	0.00	
Total daily dose of insulin (TDDI) (units)	8.10	8.30	8.50	8.10	7.50	8.40	
Minimum daily dose of insulin (units)	6.50	6.60	6.50	5.70	6.30	6.90	
Maximum daily dose of insulin (units)	8.90	9.00	9.90	9.60	10.20	9.80	
Average demand for carbohydrates (grams)	117.00	135.00	143.00	147.50	138.70	150.00	
Bolus size (daily) (units)	3.10	3.60	4.60	4.80	4.60	5.50	
Automatic (daily) base (units)	5.00	4.70	3.90	3.30	2.90	2.90	
Automatic (daily) correction (units)	-	-	0.40	0.30	0.40	0.30	
Automatic mode (SmartGuard per week) (%)	-	4	100	100	99	100	
Manual mode (per week) (%)	-	96	0	0	1	0	
Days with insulin requirement < 8 units	4	2	3	4	11	2	
Days of total daily dose of insulin < 8 units in February						Number	%
						12.00	43.00
Days of total daily dose of insulin < 8 units in March						10.00	32.00
Days of total daily dose of insulin < 8 units in April (until 23 April)						10.00	38.00
Days throughout the follow-up when the total daily dose of insulin < 8 units						25.33	37.67

## Data Availability

The authors exclude this claim because they do not have publicly archived data sets.
